# Hyperimmune anti-HBs plasma as alternative to commercial immunoglobulins for prevention of HBV recurrence after liver transplantation

**DOI:** 10.1186/1471-230X-10-71

**Published:** 2010-07-04

**Authors:** Florian Bihl, Stefan Russmann, Vanina Gurtner, Loriana Di Giammarino, Loredana Pizzi-Bosman, Martine Michel, Andreas Cerny, Antoine Hadengue, Pietro Majno, Emiliano Giostra, Damiano Castelli, Gilles Mentha

**Affiliations:** 1 Department of Gastroenterology and Hepatology, University Hospital of Geneva, Rue Gabrielle-Perret-Gentil 4, Geneva 1211, Switzerland; 2 Division of Clinical Pharmacology and Toxicology, University Hospital Zurich, Ramistrasse 100, 8091 Zurich, Switzerland; 3 Red Cross Blood Transfusion Service of Southern Switzerland, Via Tesserete 50, 6900 Lugano, Switzerland; 4 General Medicine Outpatient Clinic, University Hospital of Geneva, Rue Gabrielle-Perret-Gentil 4, Geneva 1211, Switzerland; 5 Blood Transfusion Service, University Hospital of Geneva, Rue Gabrielle-Perret-Gentil 4, Geneva 1211, Switzerland; 6 Hepatology Service, Clinica Luganese, Via Moncucco 10, 6903 Lugano, Switzerland; 7 Transplantation Unit, Department of Visceral Surgery, University Hospital of Geneva, Rue Gabrielle-Perret-Gentil 4, Geneva 1211, Switzerland

## Abstract

**Background:**

Hepatitis B immune globulins (HBIG) in combination with nucleos(t)ide analogues (NA) are effectively used for the prevention of hepatitis B virus (HBV) recurrence after liver transplantation (LT). However, associated treatment costs for HBIG are exceedingly high.

**Methods:**

Fresh frozen plasma obtained from blood donors with high anti-HBs levels (hyperimmune plasma, HIP) containing at least 4,500 IU anti-HBs was used as alternative treatment for HBV recurrence prophylaxis post-LT.

**Results:**

Twenty-one HBV-related LT recipients received HIP starting at transplantation, followed by long-term combination treatment with NA. Mean follow-up time was 4.5 years (range 0.5-12.6) and each patient received on average 8.2 HIP per year (range 5.8-11.4). Anti-HBs terminal elimination kinetic after HIP administration was 20.6 days (range 13.8-30.9), which is comparable to values reported for commercial HBIG products. All 21 patients remained free of HBV recurrence during follow-up and no transfusion-transmitted infection or other serious complication was observed. Seven patients developed reversible mild transfusion reactions. The cost for one HIP unit was US$140; average yearly HBIG treatment cost was US$1,148 per patient, as compared to US$25,000-100,000 for treatment with commercial HBIG.

**Conclusion:**

The results of this study suggest that the use of HIP may be a useful and economical approach for the prevention of HBV recurrence post-LT if used in combination with NA. Additional prospective controlled studies in larger populations are needed to confirm these results.

## Background

Without prophylactic treatment up to 80% of HBV-related liver transplantation (LT) recipients develop recurrent HBV infection after LT leading to graft damage, organ failure and increased morbidity and mortality [1-4]. Passive immunoprophylaxis with hepatitis B immune globulins (HBIG) in combination with nucleos(t)ide analogues (NA), such as lamivudine or adefovir, is highly effective for the prevention of HBV reinfection with reported HBV recurrence in only 0-10% of patients during long-term follow up after LT [5-7].

However, the costs of HBV reinfection prophylaxis with intravenous HBIG are extremely high and economic aspects become an important issue in the long-term care of these patients. The estimated cost for commercial intravenous HBIG in the peri-transplantation period range from US$50,000 to $80,000, followed by US$25,000 to $100,000 per year during long-term treatment thereafter [8-11]. Thus, over recent years efforts have been made to search for less costly regimens such as limiting HBIG treatment to 18-24 months after LT and thereafter life-long NA therapy either in mono- or bi-therapy [12-15]; or low-dose intramuscular HBIG in association with NA [16-18]. Furthermore, recent studies suggest that patients can be stratified in high and low risk for HBV recurrence based on the levels of HBV DNA prior to LT [9]. High-risk patients for HBV recurrence present detectable HBV-DNA levels at LT and might profit from high-dose long-term administration of HBIG combined with NA as earlier studies showed [10,19,20]. However, in the absence of clear data showing which patients could be suspended from HBIG, long-term HBIG therapy remains the standard of care in many centers [2].

In search of an alternative approach that would reduce costs but maintain maximum efficacy to protect from HBV recurrence, we used substituted commercial HBIG formulations fresh frozen plasma (FFP) with high anti-HBs titers (hyperimmune plasma = HIP). HIP can be easily produced in any blood transfusion center and our experience provides data on long-term efficacy, kinetics, safety and economics of HIP for the prevention of HBV reinfection after LT.

## Methods

### Patients, hyperimmune plasma administration and follow-up

In this study we report our long-term experience with 21 patients with HBV-related end-stage liver disease (ESLD) who received HIP for prevention of HBV reinfection after LT. Patients underwent liver transplantation at the Geneva University Hospital between 1989 and 2007 for HBV-related cirrhosis (n = 16), fulminant HBV (n = 3) and cirrhosis from HBV-HDV infection (n = 2) and were subsequently followed at two hepatology outpatient clinics (Geneva and Lugano) (Table 1). All patients except two were serum HBV-DNA negative at the time of transplantation. The two with detectable HBV DNA prior LT were transplanted 1989 and 1993 respectively (the HBV status of all patients is summarized in Table 1). Before 1996, patients received only HIP as recurrence prophylaxis because NA were unavailable at this time. Since 1996, combination therapy with HIP and NA (Lamivudine, 100 mg daily or Adefovir, 10 mg daily) was standard treatment for all patients. Two patients died during follow-up from non HBV-related causes. All patients except the three patients with fulminant HBV infection had chronic hepatitis B infection with HBsAg and anti-HBc for at least 6 months before evaluation for liver transplantation. With the exception of the first two patients (H1, H2), all patients with positive HBV DNA at LT evaluation were started on NA and treated while being on the waiting list in order to achieve undetectable HBV DNA at the moment of transplantation. The three patients with fulminant HBV infection were not previously known for chronic HBV infection and not vaccinated against HBV, but had a clear exposure to HBV and their serological status at time of diagnosis indicated a recent infection. All three cases however had undetectable HBV DNA at LT. The serological status of all patients before LT is summarized in Table 1.

**Table 1:** Demographic data and serological and treatment status of all patients before liver transplantation

Patient	Gender	Age at LT	Ethnicity	Year of LT	HBeAg	anti-HBe	anti-HBc	HBV DNA at LT	NA before LT	Cause for LT
H1	M	42	caucasian	1989	NA	NA	positive	qual. positive, quant. NA	none	Cirrhosis
H2	M	53	caucasian	1993	negative	positive	positive	270 pg/ml	none	Cirrhosis
H3	F	41	caucasian	1996	positive	positive	negative	undetectable	none	Fulminant HBV
H4	M	45	caucasian	1996	negative	negative	positive	undetectable	Lamivudine	Cirrhosis
H5	M	59	caucasian	1996	negative	negative	positive	undetectable	Lamivudine	Cirrhosis + HCC
H6	M	33	caucasian	1996	positive	negative	positive	undetectable	Lamivudine	Cirrhosis + HDV
H7	M	60	caucasian	1997	positive	negative	positive	undetectable	Lamivudine	Cirrhosis + HDV
H8	M	48	caucasian	1997	negative	negative	positive	undetectable	Lamivudine	Cirrhosis
H9	M	53	Hispanic	2000	negative	negative	positive	undetectable	Lamivudine	Cirrhosis + HCC
H10	M	48	caucasian	2000	negative	negative	positive	undetectable	Lamivudine	Cirrhosis
H11	F	43	caucasian	2000	positive	negative	negative	undetectable	none	Fulminant HBV
H12	M	63	caucasian	2001	negative	negative	positive	undetectable	Lamivudine	Cirrhosis + HCC
H13	F	54	caucasian	2001	negative	negative	positive	undetectable	Lamivudine	Cirrhosis
H14	M	60	caucasian	2002	negative	negative	positive	undetectable	Lamivudine	Cirrhosis + HCC
H15	M	58	caucasian	2002	negative	negative	positive	undetectable	Lamivudine	Cirrhosis + HCC
H16	M	60	caucasian	2002	negative	negative	positive	undetectable	Lamivudine	Cirrhosis + HCC
H17	M	60	caucasian	2003	negative	negative	positive	undetectable	Lamivudine	Cirrhosis + HCC
H18	M	48	caucasian	2004	negative	negative	positive	undetectable	Lamivudine	Cirrhosis
H19	M	51	caucasian	2005	positive	positive	negative	undetectable	none	Fulminant HBV
H20	F	30	african	2007	positive	negative	positive	undetectable	Adefovir+Lamivudine	Cirrhosis
H21	M	49	african	2007	positive	negative	positive	undetectable	Adefovir+Lamivudine	Cirrhosis + HCC

All patients received intra-operatively, during the anhepatic phase, at least two HIP units (range 2-10 units), each containing at least 4,500 IU anti-HBs antibodies. Postoperatively, patients received one HIP unit per day for 7 days, and then once weekly for 3 weeks post-LT. Anti-HBs serum antibody levels were measured every 4 to 8 weeks thereafter and patients received HIP on demand to maintain anti-HBs levels above 200 IU/L.

HIP was administered intravenously over a period of 1 to 3 hours in a volume of 300 ml (± 20 ml) without any pre-medication. During the transfusion all patients were closely observed, and blood pressure, pulse and body temperatures were measured twice an hour. If any allergic reaction appeared, HIP transfusions were stopped and substituted with 5,000 IU commercial HBIG. Efficacy was determined by testing the anti-HBs titer every 4-8 weeks and HBsAg and HBV DNA twice per year. Occasionally no HIP was available at the blood bank, in which case patients received 5,000 IU commercial HBIG.

The study was approved by the local Ethics Committees (in Geneva and Lugano) and the Swiss Agency for Therapeutic Products (Swissmedic protocol 2006DR2070).

### Hyperimmune plasma production, quarantine and stability

Fresh frozen plasma (FFP) derived from donors with high anti-HBs titers (>15,000 IU/L) was obtained either from whole blood donations or with plasmapheresis. Initially methylene blue for virus inactivation was added to the FFP but was discontinued in 1998 in favor of product quarantine (see below). All donors met the national criteria for blood donation (Swiss Red Cross Blood Donation Service, SRCBDS). Plasmapheresis was performed with a Haemonetics PCS2 device. From each apheresis procedure, two units of 300 ml (± 20 ml) filtered, leukocyte-deprived plasma (leuco-depletion 95%, <1 × 10^6 ^leucocytes, according to the manufacturer) were collected. Each HIP unit contained therefore at least 4,500 IU anti-HBs antibodies. All donated plasma products were screened for serological markers according to the SRCBDS guidelines including determination of anti-HCV (test introduced 1990), HCV RNA (1999), HBsAg (1974), HBV DNA (2007), anti-HIV (1985), HIV RNA (2002), Treponema pallidum (1974) and ALT levels (1989). The plasma was shock-frozen with a process that allows complete freezing within one hour to a core temperature below -30°C. After freezing, each plasma unit was stored at -80°C for at least four months but not more than 24 months for quarantine when donors were tested again for the presence of serological markers. After the second negative serological testing the HIP product was released from the blood bank for intravenous transfusion.

Some donors had persistently very high serum anti-HBs levels (>25,000 IU/L) but in others, levels dropped below the desired concentration and those received a HBV booster vaccination (Engerix-B20, GlaxoSmithKline, Belgium). Production costs for one HIP unit were estimated as US$140 (150 CHF), including the sterile single-use kit for plasmapheresis (MCS+9000 system; Haemonetics, Switzerland), two serology tests, storage costs for quarantine, screening for anti-HBs level in volunteer donors, booster vaccination and personnel expenses for donor randomization.

### Quality control and sample collections for elimination kinetics of anti-HBs after HIP administration

To evaluate whether plasmapheresis and/or storage at -80°C for 4-24 months would lead to a decrease of anti-HBs concentrations, we determined anti-HBs levels in 15 HIP units at the time of donation (in the plasma) and again at the time of transfusion (after freezing for 4-24 months for quarantine and thawing to room temperature before transfusion).

In order to determine the elimination kinetics of anti-HBs during long-term treatment with HIP, we measured anti-HBs plasma levels just before the HIP transfusion, directly after end of transfusion, and then 1 and 24 hours, and 3, 10, 14, 21 and 30 days after transfusion in 5 patients (H2, H4, H7, H15, H17). All patients were on long-term treatment with HIP and at least 4 years after LT (range 4-14 years).

### Analytical methods

Anti-HBs were determined using an enzyme immunoassay (AUSAB) with an Abbott AxSYM device (Abbott Laboratories, IL, USA). Quantitative serum HBV DNA was measured with a commercial real-time PCR kit (Taqman, Roche, Switzerland introduced 2006. Prior to 2006 a Roche Cobas Amplicore assay was used).

### Data analysis

A paired t-test was used for the comparison of anti-HBs concentrations measured at the time of donation and again at transfusion.

Calculation of anti-HBs plasma half-life after HIP transfusion was carried out by first visually controlling individual patient data in order to select only data points representing the terminal elimination phase. Anti-HBs half-life (t_1/2_) was subsequently calculated from the last 5 data points in all patients. We assumed linear kinetics, i.e. the elimination constant (k) was calculated as the slope of a linear regression line through these data points after logarithmic transformation of anti-HBs concentrations. Terminal elimination half-life was then calculated as t_1/2 _= ln2/k.

Data analyses and graphs were done using STATA version 8.2 for MacOS X (STATA Corp., College Station, TX, USA).

## Results

### Safety

Seven patients developed hypersensitivity reactions during HIP transfusions, such as hot flushes or an itching skin rash, which were treated with 2 mg clemastine iv. HIP transfusions were discontinued in these patients and they were switched for some months to commercial HBIG preparations (Hepatect^®^CP, Biotest, Dreieich, Germany). Later they were treated with HIP again but with a pre-medication of clemastine (1 mg po) two hours before transfusion, which resulted in good tolerance in all patients. These events were reported as transfusion reactions to the regional hemovigilance system. We observed no major transfusion reactions such as anaphylactic reactions or transfusion-related acute lung injury (TRALI), and no transfusion-transmitted infectious diseases.

### HIP transfusions, anti-HBs levels and efficacy

Information on follow-up, HIP administrations and anti-HBs determinations are presented in Table 2. Mean follow-up time with HIP transfusion treatment was 4.5 years (range 0.5 to 12.6 years), and the cumulative follow-up time for all 21 patients was 95.1 years. The average number of HIP transfusions was 8.2 per year (range 5.8-11.4), which corresponds to one HIP transfusion every 6-7 weeks.

**Table 2:** Follow-up time and anti-HBs levels during HIP treatment

Patient	Follow-up with HIP (years)	HIP transfusions (n)	HIP transfusions per year (mean)	HIP costs per year (USD)	Anti-HBs determinations (n)	Anti-HBs (IU/L) median (range)		Anti-HBs <200 IU/L n (%)		Anti-HBs <100 IU/L n (%)	
H1	12.6	137	10.8	1512	134	259	(38-8578)	46	(34.3)	11	(8.2)
H2	10.5	86	8.2	1148	86	351	(84-17567)	21	(24.4)	3	(3.5)
H3	5.2	34	6.5	910	51	324	(108-3555)	9	(17.6)	0	(0)
H4	9.4	65	6.9	966	82	393	(116-2130)	10	(12.2)	0	(0)
H5	11.1	125	11.2	1568	133	711	(191-3911)	1	(0.8)	0	(0)
H6	5.0	35	7.0	980	37	518	(166-1798)	3	(8.1)	0	(0)
H7	8.7	69	7.9	1106	71	454	(107-1123)	8	(11.3)	0	(0)
H8	0.5	4	8.3	1162	7	188	(76-578)	4	(57.1)	1	(14.3)
H9	1.3	10	7.8	1092	12	508	(69-1050)	1	(8.3)	1	(8.3)
H10	0.9	8	9.4	1316	21	623	(273-3016)	0	(0)	0	(0)
H11	7.2	42	5.8	812	49	560	(217-1275)	0	(0)	0	(0)
H12	2.8	21	7.5	1050	39	512	(166-2976)	3	(7.7)	0	(0)
H13	6.1	41	6.7	938	50	884.5	(101-3266)	1	(2)	0	(0)
H14	2.1	15	7.2	1008	28	829.5	(191-5048)	2	(7.1)	0	(0)
H15	1.4	10	7.2	1008	21	609	(225-2728)	0	(0)	0	(0)
H16	0.5	4	7.8	1092	7	1939	(156-5558)	1	(14.3)	0	(0)
H17	4.9	33	6.8	952	26	445.5	(95-3792)	7	(26.9)	1	(3.8)
H18	1.1	10	9.5	1330	21	597	(14-2489)	3	(14.3)	2	(9.5)
H19	2.3	16	7.1	994	30	512.5	(49-4525)	2	(6.7)	1	(3.3)
H20	0.6	7	10.8	1512	12	775.5	(63-1000)	2	(16.7)	1	(8.3)
H21	0.9	10	11.4	1596	16	721.5	(208-1000)	0	(0)	0	(0)

**All patients**	**95.1**	**782**	**8.2**	**1148**	**933**	**507**	**(14-17567)**	**124**	**(13.2)**	**21**	**(2.3)**

**Figure 1:**
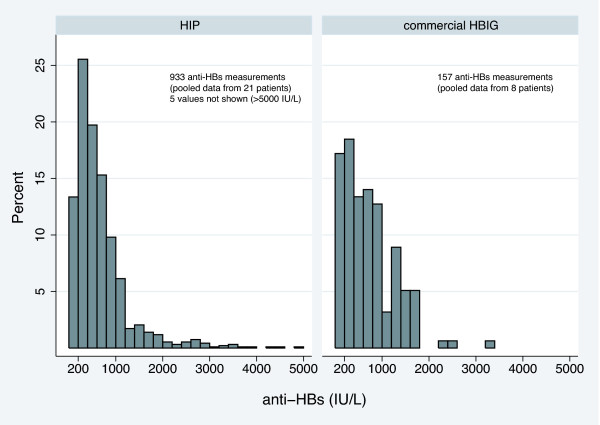
**Anti-HBs levels during treatment with HIP versus commercial HBIG**. Pooled anti-HBs levels from all 21 patients during treatment with HIP and anti-HBs levels in 8 patients when they were switched to commercial HBIG

Median anti-HBs levels, pooled from all 21 patients, during HIP treatment were 507 IU/L. Of note, these values were mostly determined shortly before the next transfusion and therefore represent trough levels rather than average concentrations during HIP treatment. Regarding the level of protection against HBV recurrence, the proportion of values below a certain lower limit such as 200 or 100 IU/L may be of interest, and is therefore presented in Table 2 and Figure 1.

Pooled anti-HBs levels during HIP treatment from all 21 patients are summarized in a histogram (Figure 1) showing their relative distribution in comparison to anti-HBs levels during treatment with commercial HBIG in eight patients who switched treatment temporarily (7 patients switched momentarily for intolerance and one patient switched for logistic reasons, see next paragraph) The proportion of measured anti-HBs values <200 IU/L and <100 IU/L during treatment with commercial HBIG was 17.2% and 7.0%, respectively, compared to 13.2% and 2.3% during HIP treatment. As reported for other HBIG formulations, we observed a pronounced inter- and intra-individual variability in anti-HBs titers under HIP treatment. And finally, all patients remained free of HBV recurrence during follow-up as indicated by negative HBsAg and HBV DNA determinations.

**Figure 2:**
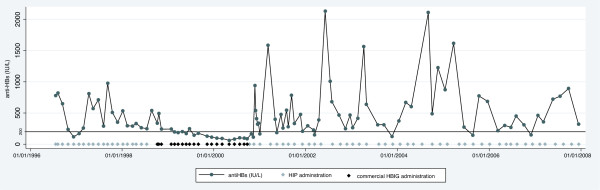
**Transfusions and anti-HBs levels in one patient who was temporarily switched to commercial HBIG and then back to HIP**. Between October 1998 and September 2000 this patient received commercial HBIG due to logistic reasons (lived temporarily far away from a transfusion center providing HIP).

Figure 2 shows anti-HBs levels and HBIG administration in one representative patient (H4), who was followed for 11.4 years after LT: this patient moved temporarily from the transfusion center providing HIP and received commercial HBIG for two years (5000 IU at each transfusion). During this time he needed 10 HBIG administrations per year, compared to an average of 6.9 HIP transfusions per year during HIP treatment. The median anti-HBs levels during HIP treatment and commercial HBIG were 393 IU/L (range 116 to 2130) and 144 IU/L (range 63 to 494), respectively. The proportion of anti-HBs values <200 IU/L and <100 IU/L during treatment with commercial HBIG were 73.7% and 31.6%, respectively, compared to 12.2% and 0%, during HIP treatment. In conclusion, annual treatment costs in this patient were considerably higher during commercial HBIG treatment (US$39,579 versus US$966 during HIP treatment) and HIP transfusions resulted in higher anti-HBs levels compared to commercial HBIG treatment (Figure 2).

### Kinetics of anti-HBs elimination

**Figure 3:**
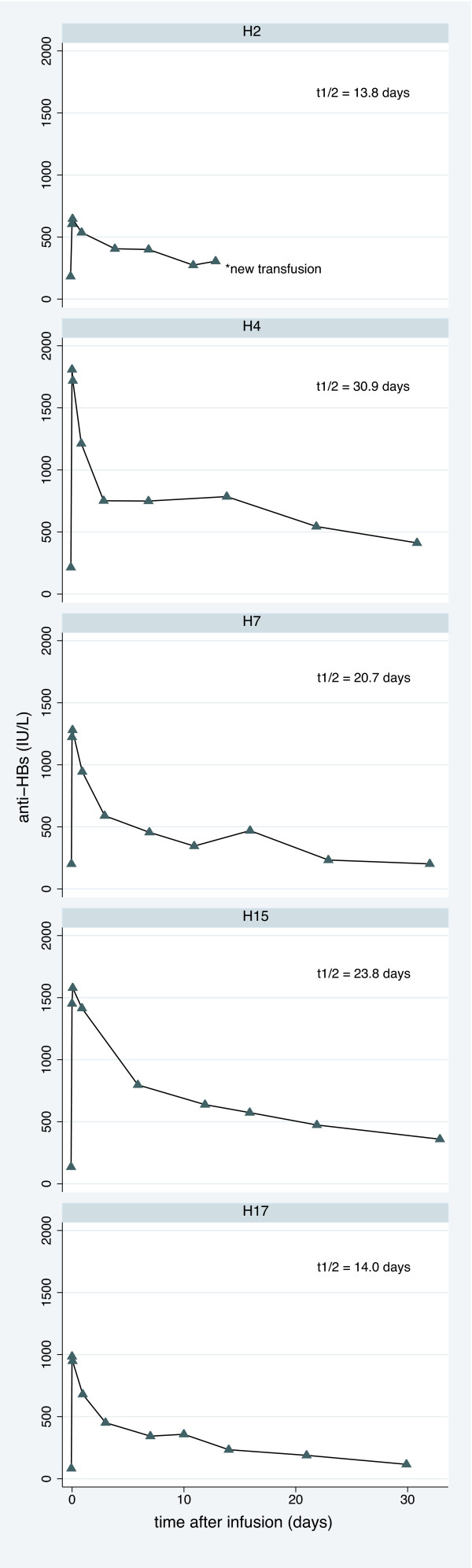
**Kinetics of anti-HBs after HIP transfusion**. Kinetics of anti-HBs levels after transfusion of one HIP unit in 5 patients during long-term HIP treatment.

Five patients participated in the study of anti-HBs kinetics. Anti-HBs plasma levels directly before and after HIP transfusions are presented in Figure 3. Mean terminal elimination half-life of anti-HBs was 20.6 days with pronounced interindividual variability (range 13.8 to 30.9 days).

### Stability of anti-HBs levels in HIP

**Figure 4:**
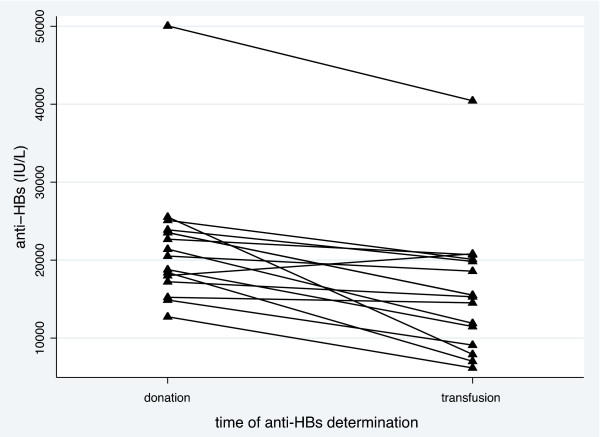
**Stability of anti-HBs levels in HIP**. Anti-HBs levels in the plasma at the time of donation and after quarantine (freezing for 4-24 months): Anti-HBs decreased significantly between donation and the time of plasma transfusion after quarantine (p < 0.001, paired t test). There was no correlation between storage time and absolute or relative anti-HBs decrease.

Anti-HBs concentrations measured in 15 HIP units at the time of donation and again at transfusion are presented in Figure 4. As shown, anti-HBs concentrations decreased in all but one sample between donation and transfusion. The mean relative concentration at the time of transfusion was 72.4% compared to the concentration at the time of donation (p < 0.001). Absolute anti-HBs concentrations were above 6,000 IU/L in all 15 tested samples with a mean of 15,959 IU/L at the time of transfusion. There was no correlation between either absolute or relative changes in anti-HBs concentrations and storage time (data not shown).

### Cost analysis

The price of one HIP unit was US$140 corresponding to the price of one fresh frozen plasma unit in Switzerland. In our experience, the average annual HBIG treatment cost per patient was US$1,148, ranking from US$812 to U$1,596 (Table 2). Thus, HIP treatment costs were notably lower than reported annual treatment costs with commercial HBIG formulations ranking between US$25,000 and $100,000 [11,21,22]. Interestingly, the HIP treatment cost was even lower than the annual cost of Lamivudine (US$2,396) or Adefovir (US$12,223) in Switzerland. Furthermore, at the time of donation, mean anti-HBs concentrations were 15,959 IU/L, or expressed as the absolute value 4,788 IU per transfused unit, which corresponds to a price of US$15 per 500 IU. This value compares to the 23-times-higher price of US$345 (370 CHF) for 500 IU commercial HBIG (Hepatect^®^) in Switzerland.

## Discussion

The introduction of treatment with HBIG was a major breakthrough for the prevention of HBV recurrence after HBV-related LT. Although there is no clear consensus on the optimal HBIG dosage and duration, it is widely accepted that HBIG plasma titers should not fall below 100 IU/L during long-term therapy. Even though earlier studies showed that higher anti-HBs levels (300-500 IU/L) seem to confer better protection against HBV recurrence, [20,23] the exceedingly high costs of commercial HBIG formulations force clinicians to search for alternatives in order to lower long-term expenses in HBV-related LT recipients. Attempts to reduce the expenses for HBIG treatment in the past include lower HBIG doses, HBIG administration by the intramuscular rather than the intravenous route, or tailored HBIG administration guided by plasma concentrations rather according to a fixed time schedule [2]. Furthermore, a recent study analyzed the long-term risk of HBV recurrence after HBIG discontinuation but continued NA therapy and found an HBV recurrence risk of 9% at 4 years after HBIG discontinuation (12). All these approaches appear to lower costs at the price of increasing the risk of HBV recurrence with consequent graft damage. In the search for new approaches with lower costs and maximum efficacy HIP has been used in some centers but previous reports included only a limited follow-up time with this approach [24,25].

This preliminary long-term experience shows that HIP treatment can achieve anti-HBs levels that are comparable to or even higher than those seen under administration of commercial HBIG. In particular, only 2.7% of measured anti-HBs levels were below 100 IU/L and HBV recurrence could be prevented in all 21 patients with an average follow-up time of 4.5 years. In addition to this convincing efficacy, patients required on average only one transfusion every 6 to 7 weeks, which is convenient and may help to maintain treatment compliance. Above all, HIP treatment achieved the important goal of substantially reducing the exceedingly high costs associated with commercial HBIG formulations. An additional advantage is that HIP can be easily produced in any blood transfusion center, where its production does not require special approval because HIP is made as FFP and therefore subject to the rigorous national safety standards that regulate blood transfusion and assure the safety of blood products. Nevertheless we realize that HIP, being a blood product, carries a residual risk for transmission of blood-borne pathogens for which donors are not screened (e.g. HEV). However, it is worth noting that in principle this also applies to commercial HBIG formulations, which likewise are human-derived blood products. The quarantine storage and repeated donor screening decrease the risk of infection with screened-for pathogens and provide the benefit of virtually no side effects, such as toxicity described for methylene blue [26]. In contrast, pathogen inactivation approaches including methylene blue or solvent detergent treatment, used for the production of commercial HBIG formulations, might be deficient for inactivation of some non-enveloped pathogens (e.g. HEV) [27]. However, in our long-term experience with a cumulative follow-up time of 95 years in 21 patients we did not detect any HIP-related blood-borne diseases. And finally, one should also consider that a high number of blood components are usually transfused during LT.

Another safety issue with HIP treatment are transfusion reactions. In our series, seven patients, i.e. 33% presented at least one transfusion-related side effect such as hot flushes or itching skin rash during long term HIP treatment. However, all reactions were mild and could be managed with antihistamines; premedication with clemastine allowed the safe use of HIP in all patients. Thus we recommend premedication for all patients who present at least one allergic reaction. We observed pronounced inter- as well as intra-individual variability in anti-HBs titers under HIP treatment, which apparently related to variability in anti-HBs concentrations in the administered HIP units. Nevertheless, the pragmatic approach of administering HIP followed by close monitoring of anti-HBs plasma concentrations but without determination of anti-HBs concentrations in the transfused units has the advantage of simple logistics and can be justified considering high anti-HBs levels that were achieved in all patients after transfusion of HIP leading to efficacious prevention of HBV recurrence. Besides, considerable variability in anti-HBs kinetics has also been described after administration of commercial HBIG formulations [11,21,28]. The calculated terminal anti-HBs half-life of 20.6 days as well as the observed variability is closely similar to what has been reported after administration of commercial HBIG formulations [28]. Finally, another benefit of HIP is that it can be used as FFP during the LT procedure, and in this way very high anti-HBs levels can be achieved during the entire operative phase without excessive costs.

On the other hand, long-term intravenous HBIG treatment may be tedious and new approaches such as low dose i.m. HBIG administration or even HBIG discontinuation are now also considered as possible alternatives. However, the administration of HIP could still be a valuable treatment for the initial intraoperative phase and the first weeks post-LT, followed by a switch to other HBIG regimens such as low dose i.m. later on.

## Conclusion

In summary, the presented results indicate that HIP in combination with an oral antiviral drug may be a valid and safe option for prophylaxis of HBV recurrence after LT. Local HIP production is an easy procedure that can be done with routine procedures at any blood transfusion center and HIP administration can dramatically reduce the exceedingly high costs associated with commercial HBIG formulations after HBV-related LT. Because of the limited number of patients in this study further studies are required in order to establish the validity of this approach.

## Abbreviations

HBV: Hepatitis B virus; LT: Liver transplantation; HBIG: hepatitis B immune globulins; HIP: hyperimmune plasma; FFP: Fresh frozen plasma; NA: nucleos(t)ide analogues.

## Competing interests

The authors declare that they have no competing interests. During this work F. Bihl was supported by a grant from the Swiss National Science Foundation (SNF/SSMBS-1240).

## Authors' contributions

FB, SR, DC and GM analyzed the data and drafted the manuscript; FB, VG, LDG and LPB collected the clinical data and SR performed the pharmacokinetic analyses and designed the figures; FB, SR, MM, EG, AC, AH and PM participated in the design and coordination of the study and contributed in data analyses and the manuscript writing and editing. All authors read and approved the final manuscript.

## Pre-publication history

The pre-publication history for this paper can be accessed here:

http://www.biomedcentral.com/1471-230X/10/71/prepub
